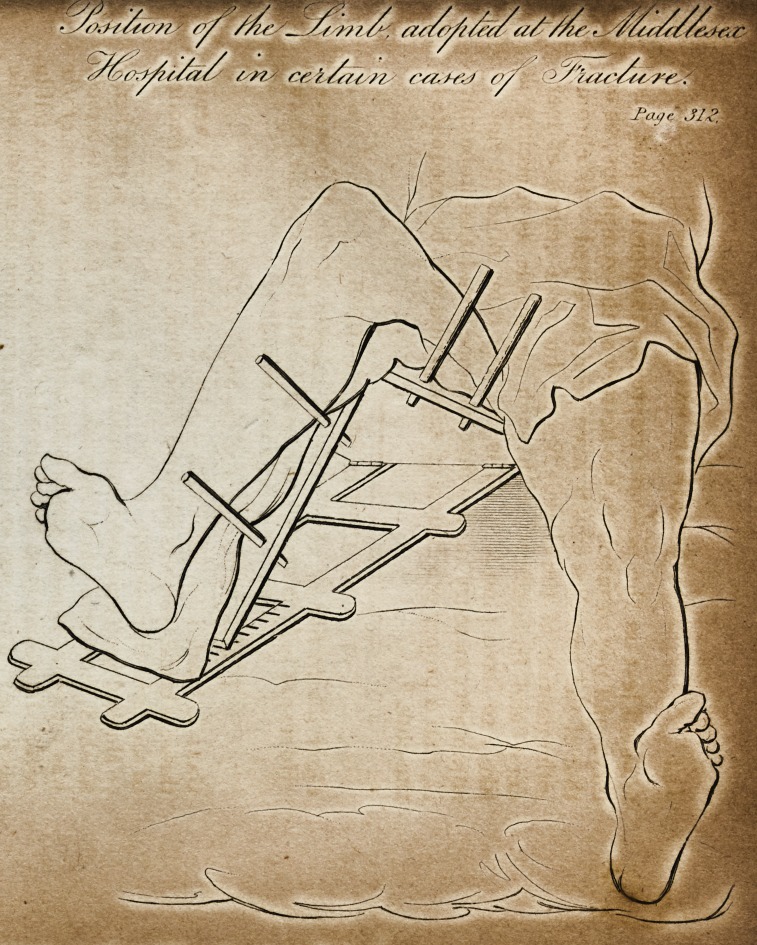# Cases Illustrative of the Treatment Adopted at the Middlesex Hospital in Fractures of the Femur

**Published:** 1826-10

**Authors:** 


### FRACTURES OF THE THIGH.

*Cases illustrative of the Treatment adopted at the* MIDDLESEX HOSPITAL *in Fractures of the Femur,* by Mr. BELL and Mr. SHAW.

[WITH AN ENGRAVING.]

### I. *Oblique Fracture of the Shaft of the Femur.*

CASE I. *treated by* Mr. BELL.—Joseph Shelston, aged twenty-three, a bricklayer, was admitted June 30th, 1826, with a fracture of the right thigh-bone.

He had been employed in building a vault, and, a part being finished, the frame-work, on which the arch of the vault is turned, was removed, in order (as it is termed) *to strike the centre of the arch.* Immediately on the removal of the support, the arch fell in upon him. There was an oblique fracture about the middle of the shaft of the femur. The soft parts were but little injured, and the swelling which supervened was slight; the shortening of the limb was considerable. Both limbs were placed over the double inclined plane; the fractured bone was reduced; an evaporating lotion was applied to the thigh; and a purge of calomel and jalap was administered.

In a few days the tumefaction had subsided, and now the relative situations of the fractured portions became more evident. The upper part was felt projecting outwards in a sharp point, and the lower division of the fracture had been again retracted about an inch. The thigh was extended, and splints and a bandage were applied. On visiting him next morning, the above displacement of the extremities of the broken bone had recurred; and, although care was taken to replace them, still, on each returning day, the bones were found to have resumed their former position.

In order to correct this projection of the upper part of the fracture, caused by the action of the gluteal muscles, the fractured limb was placed on a double inclined plane, similar to that represented in the *engraving;* and the leg and foot were carried outwards, so as to bring the lower portions of the fractured bone in a line with the upper. The whole limb resting on the inclined planes, thus formed an obtuse angle with the trunk of the body. By this

Mr Travers's Case of Wound of the Carotid Artery.

Page 339.

Position of the Limb, adopted at the Middlesex Hospital in certain cases of Fracture.

Page 312.

Engraved for the London Medical & Physical Journal, Oct. 1, 1826.– New Series, No. 4.

means the fractured bone was kept perfectly straight, and the broken extremities were retained in apposition. Thus the limb preserved its proper length, and shortening was effectually prevented.

He was discharged cured August 28th, at which time there was not the least shortening, or other defect of the limb. Indeed, it was impossible for the bystander to distinguish which thigh had been fractured.

Case II. *treated by* Mr. Shaw.—George Crump, aged fifteen, was brought to the hospital July 9th, 1826. In attempting to jump over a hedge, he had fallen, and broken his right thigh-bone. It was fractured rather obliquely, near its middle. There was great distortion and shortening of the limb; the knee fell so much inwards, that the thigh formed in itself a complete semicircle.

The fractured portions were replaced, and both extremities were put upon the double inclined plane, as in the former case; but with the like unfavourable result: for, as soon as the tumefaction had diminished, the upper part of the fractured femur might be distinctly felt projecting outwards. All attempts to retain the broken extremities of the bone in their proper situation were futile, until the position of the limb adopted in the former case was had recourse to. The leg and lower portion of the fractured femur were carried in a direction outwards, and then placed on the double inclined plane already noticed: thus the extremity was made to form an angle with the trunk of the body, as before described.

This position was quite effectual: the thigh was rendered perfectly straight, and the broken ends of the bone were brought into accurate coaptation, and were without difficulty thus retained. He was discharged August 28th, 1826. There was no perceptible difference in the two thighs, either as regarded their length or straightness.

The acknowledged difficulty attending the successful treatment of fractures of the thigh-bone, has led to the recital of the above cases. Indeed, the deformity which so frequently accompanies the union of the broken femur has, with much reason, been considered as an opprobrium to surgery.

There can be no better proof of the difficulties of a subject, than the employment of a variety of means to overcome them; and it is a fair inference, that, whilst such various means continue to be employed, the opposing obstacles remain still unsubdued.

The numerous methods resorted to in the treatment of fractures of the femur, are well known. Indeed, so various and opposite are they, that it is obvious the correct principle is not yet generally understood: for, among our first surgeons, few can be found who agree as to the most successful method of cure. No better exemplification of the truth of this statement can be afforded, than the practice in our public hospitals.

That excellent surgeon, POTT, was the first to point out the importance of relaxing the muscles of a fractured limb. He showed that want of success in the treatment of fractures was attributable to neglect of this principle; and that a well-judged position of the limb would greatly conduce to a proper union.

This rule, of relaxing the muscles of the limb by position, may be considered of the greatest value in the treatment of fractures of the shaft of the femur: for it is acknowledged that the shortening and deformity, which are but too often the results of this accident, are produced by the numerous strong and powerful muscles which move this bone.

The obvious principle, therefore, to be attended to in the treatment of the fractured thigh bone, is to place the injured limb in that position which most effectually relaxes these muscles; and the double inclined plane appears to answer this intention better than any other form of apparatus as yet proposed.

The distortion consequent on fracture of the femur, depends upon the upper portion of the fractured bone being displaced by the action of the strong muscles which are inserted into it. This displacement occurs in a direction upwards and outwards, the gluteal muscles dragging it in the latter direction, whilst the psoas and iliac elevate and rotate it outwards. It will also be perceived that these muscles must act with greater force and advantage when the fracture is high in the shaft of the femur; and, consequently, that the projection outwards, and the elevation, will be increased in proportion as the bone may be broken high up and near the trochanters.

From the above cases, it appears that the success attending the employment of the double inclined plane, in fractures of this part of the bone, may be rendered still more perfect by further attention to position, and by a slight alteration in the apparatus. Instead of the double inclined plane for both extremities to rest upon, an apparatus fitted for one only will be necessary: for, by this contrivance, we have it in our power to give to the limb whatever direction, with relation to the trunk, we may think proper.

These cases also show that, when a fracture occurs in any part of the shaft of the femur, *(unless it be within two or three inches of the trochanters,)* placing the injured limb on this double inclined plane, in the position alluded to, prevents any chance of the extremity being shortened or distorted. This position consists in the inclination of the thigh and leg outwards, so that they may form an obtuse angle with the trunk of the body. By these means all the muscles will be relaxed; the lower portion of the fractured bone will be brought into a line with the upper; the broken extremities will be retained in due apposition; there will be no further tendency to displacement, and thus shortening of the limb will be prevented.

Although we have no cases at present to illustrate this plan of treatment in fractures of the upper part of the thigh-bone within a few inches of the trochanters, still, from the recollection of cases,—from the numerous preparations of fracture at this part of the femur which we have seen,—and from the delineations of similar preparations in Sir ASTLEY COOPER'S work* on Dislocations and Fractures, and in Mr. BELL'S work on Injuries of the Spine and Thigh-bone,† it is evident that the fracture at this part of the femur is, of all others, the most liable to be followed by the elevation and dragging out of the upper portion; and this to so great an extent as to threaten the most serious distortion, and even to render the patient lame for life.

H. S., a countryman, applied at this hospital, a few weeks since, for advice, having suffered from a fracture of this kind. The upper part of the fractured bone projected outwards in a most remarkable degree, and it overlapped the lower, so that nearly the whole diameter of the cylinder of the upper portion might be felt. In the treatment, both extremities had been placed over the double inclined plane, and the fractured thigh had been supported by bandages and splints. Although six months had elapsed since the occurrence of the accident, he remained still so weak in the limb as to be unable to walk without a stick. This case shows the inefficacy of the common double inclined plane for both limbs, in the treatment of this fracture.

In a case of this kind, the injured limb may be placed on the double inclined plane here described, the direction of which outwards must, for reasons already stated, be increased, and the trunk of the body must be elevated. By such position, the gluteal, psoas, and iliac muscles will be relaxed, and distortion prevented.

The additional support afforded by bandages and splints is in all cases necessary.

The Engraving shows the position of the limb alluded to in the foregoing cases. It will be observed that the inclined

* Plate XII. fig. 6. † Plate VII. fig. 1.

planes, being of equal length, and resting on the same level, must have the same inclinations. At the angles under the ham and the nates, there are hinges; and the part under the heel is moved on a rack, formed in the lower frame-work. Thus the angle formed by the planes may be increased or diminished at pleasure.* The difference between this apparatus and the fracture-box of PETIT, is at once manifest. The inadequacy of the latter to answer the ends intended, must be equally evident; and it is only a matter of astonishment how the two could ever have been confounded.

II. *Transverse Fracture of the Condyles of the Femur.*

CASE III. *treated by* Mr. BELL.—James Soundy, a strong athletic man, about fifty-six years of age, was admitted into the Middlesex Hospital, June 8th, 1826. He had received a kick from a horse, on the upper and inner part of the right leg, just below the head of the tibia. He was brought to the hospital shortly after the occurrence of the accident.

There was much tumefaction and swelling about the knee-joint and condyles of the femur, and too great an extent of lateral motion between the bones of this part. This was found to depend upon a transverse fracture of the femur, immediately above its condyles. A crepitus might be easily felt in this situation.† The knee-joint appeared to have received but little injury further than what must necessarily have arisen from the straining of its ligaments, and from the contiguity of the fracture.

The thigh and leg were placed in the *straight position*, and confined by junks. Leeches were applied to the knee, and afterwards an evaporating lotion. He was well purged with calomel and jalap.

Much inflammation about the knee-joint followed, which was actively combated by local depletion. The parts about the joint became much thickened: this was evidently caused by an extensive deposit of coagulable lymph. As soon as the inflammation was sufficiently subdued, bandages and splints were applied.

Passive motion was attempted about the fourth week, but so much pain and heat were induced, that it became necessary to discontinue it. When this excitement had subsided, a stimulating liniment was applied. Passive motion was afterwards employed, and he was discharged August 1st, having regained considerable motion in the joint.

* There is a provision for fitting a foot-board to the apparatus. By this contrivance, the foot is easily fixed; and, while this assists in keeping up a certain degree of extension, it at the same time steadies the whole limb. To have put it in the drawing, would have rendered the whole less distinct.

† The crepitus communicated in fractures of the extremities of the long bones is peculiar, depending upon the cancellous structure of these parts. It is not easily described, but is soon recognised after being once felt.

He called at the hospital September 7th, the use of the joint being then nearly perfect.

Case IV. *treated by* Mr. Bell.—Maria Banfield, aged twenty-three, was brought to the Middlesex Hospital, on the 24th July, 1826. Whilst carrying a pail of water down stairs, she slipt, and fell. The right knee and leg were bent under her, and received the whole weight of the body.

There was found to be a transverse fracture just above the condyles of the right femur, which communicated a crepitus as peculiar as that in the former case, although less distinct. Fifteen leeches were immediately applied, and afterwards an evaporating lotion. The bowels were freely opened; and the limb was placed in a *straight position.* Little swelling or tumefaction followed, so that in a few days the splints and bandage were applied, which retained the fractured limb perfectly straight, and without motion.

In the fourth week, passive motion was begun; and she was discharged August 29th, with the motion of the joint completely restored, and with perfect power over the limb.

It may be observed that, in this class of cases, the fracture being low down, the powers which tend to distort the upper part in the fractures of the shaft are comparatively weak; and that the broad opposing surfaces presented by the fractured extremities of the bone must be effectual in preventing displacement. In addition, it may also be remarked, that, if this fracture were placed on the double inclined plane, the weight of the leg and foot would pull down and rotate the condyles, and thus displace the lower portion of the fracture.

It is unnecessary to add any cases of fracture of the neck of the femur, (although two have recently occurred at this hospital,) because the plan of placing both extremities over the double inclined plane, and swathing the pelvis and trochanters with a bandage or belt, is now generally adopted The patient is to be kept in this position for six weeks at least, as it is next to impossible to decide before-hand whether such a fracture be so circumstanced as to admit of union or not.

---

## Figures and Tables

**Figure f1:**